# Brain Connectivity Estimation Network for the Identification of Dementia

**DOI:** 10.3390/brainsci15090975

**Published:** 2025-09-10

**Authors:** Ji Xi, Zhengwang Xia, Weiqi Zhang, Li Zhao

**Affiliations:** 1 School of Computer Information Engineering, Changzhou Institute of Technology, No. 666, Liaohe Road, Changzhou 213022, China; xiazhengwang@czust.edu.cn (Z.X.); zhangwq@czust.edu.cn (W.Z.); 2 School of Information Science and Engineering, Southeast University, Nanjing 210096, China; zhaoli@seu.edu.cn

**Keywords:** brain connectivity, neurological disorder, causal discovery, dementia recognition

## Abstract

**Objectives**: The brain network serves as a reliable tool for diagnosing neurological disorders. However, the current modeling algorithms for brain networks often rely on several assumptions regarding the interactions between brain regions, which can be inaccurate. For instance, some studies assume linear relationships among brain regions. Additionally, some research suggests that certain brain regions do not significantly influence outcomes when assessing directional influence between paired regions. **Methods**: To address this issue, we introduced a novel method for modeling brain connectivity structures that estimates interactions among regions from a different perspective. More importantly, this method considers all the relevant brain regions during evaluation rather than isolating individual relationships. **Results**: To validate its effectiveness, we conducted extensive experiments using publicly available datasets. The proposed method achieved superior performance across all tasks. **Conclusions**: The results demonstrate that our method not only excels in identifying various brain disorders but also uncovers new biomarkers, providing fresh insights into neurological disorder research.

## 1. Introduction

Alzheimer’s Disease (AD) affects millions of people worldwide and has emerged as a significant public health concern [1,2]. Despite extensive research efforts, an effective clinical treatment for AD remains elusive. Mild cognitive impairment (MCI) serves as a transitional stage between the normal cognitive decline associated with aging and the more severe decline observed in dementia. Numerous studies indicate that individuals with MCI are at an increased risk of developing AD, with over half likely to progress to AD within five years [3,4]. Clinical trials have shown that early interventions, such as medication and cognitive exercises, can moderately delay the progression of MCI, thereby reducing the risk of developing AD [5]. Consequently, accurately diagnosing MCI has attracted considerable attention and has become a central focus of academic research in recent years. However, differentiating MCI from normal aging poses challenges due to their subtle distinctions [6].

Functional magnetic resonance imaging (fMRI) is a non-invasive technique that has demonstrated significant potential in diagnosing neuropsychiatric disorders, providing robust support for the detailed analysis of brain activity [7]. Research indicates that functional brain networks can capture the temporal correlations between different brain regions, aiding in the identification of areas closely associated with functional impairments resulting from neurological conditions [8,9]. In the analysis of brain imaging, several modeling approaches for brain networks have been introduced, which can be categorized into three types: Pearson correlation (PC) brain networks [10], high-order brain networks [11], and sparse brain networks [12]. Specifically, PC-based methods, which are widely employed to estimate functional brain networks, assess the interaction strength between brain regions by calculating the correlation of Blood Oxygenation Level-Dependent (BOLD) signals. However, PC-constructed brain networks often exhibit high density, complicating the accurate identification of disease-relevant connections [13]. As an advancement over PC methods, high-order brain networks also tend to generate dense graph structures. In contrast, sparse brain network methods can more effectively highlight strongly correlated interaction patterns by excluding weakly correlated connections. Studies have demonstrated that sparse brain networks are more efficient in identifying brain disorders [14,15].

However, it is essential to recognize that the aforementioned methods are limited to defining interactions between brain regions solely through correlation. A significant drawback of these approaches is their inability to ascertain the directional influence between brain areas. Given the brain’s exceptional complexity as a neural system, the internal transfer of information heavily depends on neuronal activity and connectivity patterns [16]. This highlights the necessity for a brain network modeling framework that incorporates directional factors to accurately represent interaction patterns among brain regions. Considering the importance of directional influence in understanding brain organization, many directionality-based methods are receiving increasing attention from researchers in brain network modeling as they facilitate the clear identification of directional influences between brain regions. Advances in this field have led to the development and application of various directionality-based algorithms, with Granger causality (GC) being a prominent example [17].

**Figure 1:**
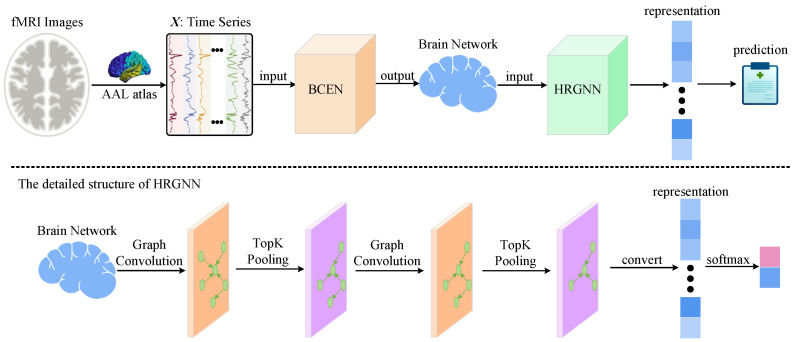
Illustration of the proposed framework for dementia identification. Initially, signals from each brain region are extracted using the Automated Anatomical Labeling (AAL) atlas. Subsequently, the extracted time-series data, referred to as X, is then input into the BCEN model to estimate the directional influences among brain regions, resulting in the generation of a brain network as output. Ultimately, the estimated brain network is fed into the hierarchical representation graph neural network (HRGNN) to obtain the prediction results.

To address the limitations of the current dementia recognition methods, we present a novel framework that we named the Brain Connectivity Estimation Network (BCEN) to estimate the directional influences among various brain regions, as illustrated in Figure 1. Furthermore, to improve recognition accuracy for brain disorders, we have integrated a widely used graph pooling layer to develop hierarchical representations of brain networks. Tests conducted on the Alzheimer’s Disease Neuroimaging Initiative (ADNI) database demonstrate that our method surpasses existing techniques. In conclusion, our framework offers several significant advantages:

- The proposed BCEN is a flexible approach for inferring directional influences between brain regions.
- The employed graph pooling layer effectively captures critical substructures and helps to enhance the identification performance of neurological disorders.
- The estimated brain network generated by our method exhibits nonlinear interactions between brain regions, surpassing traditional linear approaches.

The layout of this paper is organized as follows: Initially, in Section 2, we examine the most relevant techniques associated with this research. Subsequently, Section 3 outlines the method for estimating brain networks and the guidelines for updating the graph pooling layer. Then, Section 4 describes the materials and procedures involved in training the model. Section 5 and Section 6 present the experimental results and conclusions of our study. Lastly, Section 8 summarizes the research findings.

## 2. Related Work

In this section, we begin by outlining the latest developments in estimating brain networks in Section 2.1. Subsequently, we examine the advancements achieved by graph neural networks (GNNs) in identifying neurological disorders in Section 2.2.

### 2.1. Causality for Brain Network Estimation

Granger causality and its modified versions are powerful tools for identifying directional influences between variables [18]. The fundamental assumption is that, if historical data from one time series, X, improves the prediction of future values in another series, Y, then X is considered the Granger cause of Y. Over the decades, numerous researchers have utilized Granger causality and its derivatives to investigate interactions between brain regions, leading to significant findings. For example, Su and colleagues developed an advanced nonlinear Granger causality method to map brain networks [19]. Their research revealed that patients with amnesic mild cognitive impairment exhibited significant declines in global efficiency and average clustering coefficients of their brain networks compared to healthy subjects. Yi et al. employed a novel approach known as nonparametric Granger causality to evaluate time-evolving directional influences between brain regions [20]. They identified distinct brain activity patterns between individuals with left and right hemiplegia.

In addition to GC, several alternative methods are employed to infer effective connectivity between brain regions. Partial Directed Coherence (PDC) is a frequency-domain Granger-causality-based approach that evaluates directional influences among multivariate time series by extending the concept of partial coherence [21]. Similarly, the Directed Transfer Function (DTF) quantifies the directional influences between brain regions in the frequency domain, utilizing a normalized version of the multivariate autoregressive (MVAR) model coefficients to enhance interpretability [22]. Transfer Entropy (TE), an information-theoretic measure, assesses the directed transfer of information between two time series by quantifying how knowledge of past states in one system reduces uncertainty about future states in another, making it particularly suitable for capturing nonlinear interactions [23]. Dynamic Causal Modeling (DCM) employs a Bayesian framework to infer effective connectivity by explicitly modeling how neural activity in one region causally influences another under experimentally controlled conditions, providing mechanistic insights into neural dynamics [24]. Finally, Dynamic Bayesian Networks (DBNs) extend static Bayesian networks to model time-varying probabilistic dependencies between brain regions, incorporating hidden Markov models (HMMs) or state-space models to capture dynamic connectivity changes across brain regions [25].

However, when inferring directional influences between brain regions, the aforementioned methods generally rely on implicit assumptions about the input data, which may limit their applicability and the reliability of the results. For instance, models such as PDC, DTF, and DCM are all based on linear system theory, implicitly assuming that the input time-series data satisfy conditions such as stationarity and linear additivity. If the actual data do not meet these assumptions, systematic biases may arise, leading to erroneous identification of spurious connections. In particular, DCM’s methodology centers on characterizing the causal mechanisms of neural activity through generative models (such as neural mass models). This process not only requires the pre-specification of precise neural dynamic equations but also demands a deep understanding of the biophysical significance of model parameters. Consequently, this imposes high demands on researchers’ prior knowledge and model tuning experience, potentially limiting its general applicability in exploring neural phenomena. To address these challenges, we have developed a novel generative adversarial network (GAN) designed to estimate directional influences among brain regions. This network incorporates all relevant factors for a comprehensive analysis.

### 2.2. Graph Neural Network for Brain Disorder Identification

Brain networks depict the interplay among different brain regions in the form of graphs, forming a highly interconnected system. Given the substantial benefits of graph neural networks (GNNs) in handling reasoning tasks involving graph-based data, various GNN models have been devised and utilized in identifying neurological diseases. In particular, Zhang and colleagues designed an innovative GNN to improve the performance of traditional recognition frameworks by integrating additional clinical data [26]. This model achieved outstanding classification results on public datasets. Zheng et al. introduced an interpretable GNN model for identifying neurological disorders, demonstrating exceptional performance on both simulated and real-world datasets [27].

Several studies have demonstrated that neurological disorders primarily lead to localized disruptions in brain function rather than uniformly affecting the entire brain [11,12,15]. However, the current graph neural network (GNN)-based classification frameworks overlook the significance of these localized effects in their design. Therefore, it is essential to accurately identify the substructures of brain networks that are most closely associated with neurological disorders. To address this limitation, we have integrated graph pooling operations into our framework to obtain hierarchical representations of brain networks, thereby improving the recognition accuracy of subsequent tasks.

## 3. Methodology

In this section, we first introduce the approach for estimating brain networks in Section 3.1. Subsequently, we explain the updating mechanism for learning hierarchical graph representations in Section 3.2.

### 3.1. Brain Structure Inference

As mentioned in Section 2.1, the traditional algorithm imposes many constraints on the input time-series data when analyzing directional influences among brain regions. To address the limitations of the traditional Granger causality method, we introduce a new Brain Connectivity Estimation Network (BCEN) that can estimate directional influences among all brain regions while concurrently considering all relevant factors.

**Figure 2:**
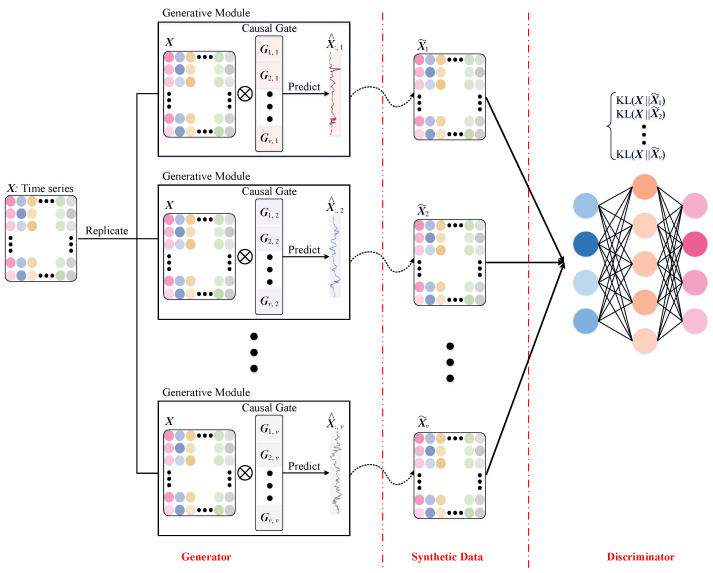
The detailed structure of BCEN. First, the time-series data X is input into the generative module to identify the directionality associated with the corresponding brain region. Next, each column of the time-series data X is replaced with the estimated signal, denoted as ${\tilde{X}}_{i}$. Finally, a discriminator network is utilized to distinguish between real samples and synthetic samples.

The specific architecture of BCEN is illustrated in Figure 2, which consists of multiple generative modules that evaluate directional influences among brain regions using time-series data $X\in R^{t\times v}$. Here, *t* represents the length of the time points, and *v* denotes the number of brain regions. In the domain of causal inference, it is widely accepted that causes precede effects. Based on this principle, the directional influence of other brain regions on the *i*-th brain region can be expressed as follows:

(1) $${\hat{X}}_{:,i}=\sigma \left(\sum_{m=1,m\neq i}^{v}G_{m,i}X_{:,m}\right)$$

where the symbol σ(·) is a nonlinear function, and $G_{m,i}$ denotes the directional influences of the *m*-th brain region on the *i*-th brain region. *v* equals the number of brain regions.

To assess the directional influences among all brain regions, we have developed an innovative generative network composed of *v* generative modules. The *i*-th module, denoted as $f_{i}$, includes a causal gate $G_{:,i}\in R^{v}$, which is designed to retain the directional influences from all other brain regions on the *i*-th brain region. The generator network, consisting of *v* modules, facilitates the simultaneous inference of directional influences among all brain regions.

For the *i*-th generative module, we can obtain the estimated BOLD signal for the *i*-th brain region, denoted as ${\hat{X}}_{:,i}$. A synthetic sample, ${\tilde{X}}_{i}$, is generated by replacing the actual BOLD signal of a specific brain region, $X_{:,i}$, with its predicted signal, ${\hat{X}}_{:,i}$. Since the generator network comprises *v* generative modules, each individual can produce *v* synthetic samples through one-to-one substitution, specifically ${\tilde{X}}_{1},{\tilde{X}}_{2},\ldots ,{\tilde{X}}_{v}$.

To enhance the realism of the predicted Blood Oxygenation Level-Dependent (BOLD) signals, denoted as $\hat{X}$, in comparison to the actual signals, represented as X, we integrated a discriminative network. This approach guarantees that, even after extensive training, the model is unable to consistently differentiate between synthetic and authentic samples. The loss function of the BCEN can be formulated as follows:

(2) $$\begin{array}{c} G_{loss}=\left|\right|\hat{X}-X{\left|\right|}_{2}^{2}+\frac{1}{v}\sum_{i=1}^{v}C_{:,i}\\ D_{loss}=\sum_{s=1}^{v}D_{KL}\left(X|\right|{\tilde{X}}_{i}) \end{array}$$

where $G_{loss}$ is the generator loss, and the introduction of the sum of directional influences $C_{:,i}$ aims to achieve optimal fitting performance while maintaining low complexity. $D_{loss}$ denotes the discriminator loss, which is used to minimize the differences in data distribution between the synthetic sample ${\tilde{X}}_{i}$ and the real sample X by reducing the Kullback–Leibler divergence.

### 3.2. Hierarchical Graph Representation Learning

Graph neural networks (GNNs) have garnered significant attention for their exceptional ability to model graph-structured data. Numerous studies indicate that obtaining multi-level representations can enhance the effectiveness of subsequent tasks. Consequently, we introduced a novel GNN called the hierarchical representation graph neural network (HRGNN), which is designed to derive hierarchical representations from brain networks. Figure 1 illustrates the HRGNN architecture, which consists of three graph convolution layers and three graph pooling layers.

The graph convolution operation for the *k*-th layer is defined as follows:

(3) $$N^{k+1}=\sigma \left({\tilde{D}}^{-\frac{1}{2}}{\tilde{G}}^{k}{\tilde{D}}^{-\frac{1}{2}}N^{k}W^{k}\right)$$

where ${\tilde{G}}^{k}$ represents the subgraph of the brain network G after k−1 layers, and $N^{k}$ denotes the node feature matrix subsequent to k−1 layers. Both ${\tilde{G}}^{k}$ and $N^{k}$ serve as inputs to the *k*-th graph convolution layer. The function σ(·) is a nonlinear activation function. Additionally, $\tilde{D}$ is the diagonal degree matrix of ${\tilde{G}}^{k}$, and $W^{k}$ is a trainable weight matrix.

The objective of the graph pooling process is to preserve the crucial structure of the graph while reducing its size. In this context, we utilize the widely adopted TopK pooling method to facilitate subsequent operations [28]. The formulation for graph pooling in the *k*-th layer can be expressed as follows:

(4) $$\begin{array}{c} index=\mathrm{TopRank}(N^{k},p)\\ G^{k+1}=G_{index,index}^{k} \end{array}$$

where the function TopRank(·) returns the indices of the top *p* nodes according to node importance. $G^{k+1}$ is the updated brain network following the *k*-th graph pooling layer.

Ultimately, the representations of various subgraphs are combined to create a unified representation for diagnosing dementia in patients. The aggregation rule is expressed as follows:

(5) $$\begin{array}{c} G_{r}=r_{1}\;||\;r_{2}\;\left|\right|\;r_{3}\\ r_{i}=\left|\right|N^{k}{\left|\right|}_{1} \end{array}$$

where $r_{i}$ denotes the *k*-th subgraph representation, and || represents the concatenation operation. The notation $\left|\right|\cdot {\left|\right|}_{1}$ indicates the computation of the $L_{1}$ norm on a row-wise basis. The aggregated graph representation $G_{r}$ is subsequently input into a Multi-Layer Perceptron (MLP) layer with a softmax classifier for graph classification. The cross-entropy loss function is employed for training the HRGNN.

## 4. Experimental Setup

In this section, we first present the demographic information of the dataset used in this study in Section 4.1, followed by the training details of BCEN and HRGCN in Section 4.2.

### 4.1. Data and Preprocessing

For this study, we utilized resting-state fMRI data from 214 participants obtained from the publicly accessible Alzheimer’s Disease Neuroimaging Initiative (ADNI) database (https://adni.loni.usc.edu, accessed on 31 August 2025). The participants included normal controls (NCs), individuals with early mild cognitive impairment (eMCIs), and those with late mild cognitive impairment (LMCIs). Demographic details are presented in Table 1. All human subjects provided written informed consent after receiving a comprehensive explanation of the study, both in writing and verbally. The research received approval from the ADNI Research Ethics Board (https://adni.loni.usc.edu/data-samples/adni-data/study-cohort-information/, accessed on 31 August 2025).

During the preprocessing of fMRI data, we adhered to standard protocols, which are detailed below. Initially, we removed the first 5 volumes from each participant to minimize noise, retaining 135 volumes for further analysis. The functional images were then aligned to the first image and transformed into Montreal Neurological Institute (MNI) space, with a voxel size of 3 × 3 × 3 mm^3^. Subsequently, we utilized the Conn Toolbox (https://web.conn-toolbox.org/, accessed on 31 August 2025) for various tasks, including direct segmentation, normalization, linear detrending, and functional smoothing. Ultimately, we extracted time-series data for each brain region from the preprocessed images, using the AAL atlas as a reference.

### 4.2. Model Training

For the BCEN, each generative module $f_{i}$ is configured as a one-hidden-layer neural network with 100 tanh units. The causal gate $C_{:,i}$ is randomly initialized, except for the self-loop terms $C_{i,i}$, which are set to 0. The shared discriminator network is designed as a two-hidden-layer neural network with 100 hidden ReLU units. The Adam optimizer, with a learning rate of 0.001, is employed to optimize the model. The generator network is trained for 3000 epochs, while the discriminator network is trained for 1000 epochs.

For the HRGNN, the Adam optimizer is utilized to optimize the network. The learning rate is set to 0.001, and the model is trained for 1000 epochs. The pooling parameter *p* in Equation (4) is explored within the range of {0.1,0.2,…,0.9}. The MLP consists of two fully connected layers, with the number of units in each layer set to 64 and 32, respectively, followed by a softmax classifier.

## 5. Results

In this section, we provide a comprehensive explanation of the comparison techniques introduced in Section 5.1. Following this, we present the experimental results in Section 5.2. Finally, we conduct an ablation study in Section 5.3 to demonstrate the effectiveness of our proposed approach.

### 5.1. Comparison Methods

In this paper, we design two neural networks: BCEN and HRGNN, used to advance the traditional framework for recognizing neurological disorders. BCEN is employed to evaluate directional influences among brain regions, thereby enhancing conventional brain network modeling techniques. Conversely, HRGNN represents an improved graph neural network (GNN) that can derive multi-level representations of brain networks, signifying a significant advancement over traditional classifiers.

To evaluate the effectiveness of our proposed brain network modeling algorithm, BCEN, we conducted a comparative analysis against nine widely used brain network modeling algorithms: (1) Pearson correlation (PC) [8], (2) sparse brain network (SBN) [29], (3) low-rank brain network (LRBN) [12], (4) sparse low-rank brain network (SLRBN) [12], (5) Granger causality (GC) [30], (6) multivariate Granger causality (mGC) [17], Partial Directed Coherence (PDC) [21], Directed Transfer Function (DTF) [22], and Transfer Entropy (TE) [23]. The first four methods define interactions between brain regions based on correlation, while the latter two, along with our BCEN, analyze these interactions from the perspective of directionality. In this study, the classifier utilized the weights of brain network edges as features to generate predictive results.

To evaluate the performance of the advanced graph classifier HRGNN in identifying dementia, we compared it with two widely used classifiers: Random Forest (RF) [31] and Support Vector Machine (SVM) [32]. The RF was configured with 500 trees and a maximum depth of 3. For the SVM, we set 200 iterations, employed a linear kernel, and used a regularization coefficient of 1.0. The pooling parameter *p* for HRGNN was determined to be 40, with the rationale detailed in Section 5.3.

To evaluate the effectiveness of various algorithms in identifying dementia cases, we employ four widely used metrics: accuracy (ACC), sensitivity (SEN), specificity (SPE), and the F1 score. To enhance the reliability of the classification results, we implement a 10-fold cross-validation method.

### 5.2. Experimental Results

Table 2 presents the recognition performance of all the methods across three classifiers. Additionally, we conducted a two-sample *t*-test to compare the results generated by the proposed method with those obtained from suboptimal approaches. Statistically significant differences between the two sets of results are indicated by an asterisk (*) placed in the upper right corner of the corresponding values. Our proposed method consistently achieves superior classification accuracy in most scenarios, demonstrating its effectiveness in estimating directional influences among brain regions. Furthermore, two key observations warrant attention. First, among the four correlation-based methods, LRBN often produces the best results, likely due to the integration of prior information [12]. In contrast, SLRBN frequently underperforms, probably because it imposes numerous constraints that lead to an overly sparse estimation of the brain network, thereby excluding valuable features. Second, directionality-based methods generally outperform correlation-based methods, achieving accuracy levels exceeding 80% in many instances. This suggests that directionality-based methods are more adept at estimating interaction relationships among brain regions, potentially due to their ability to eliminate spurious connections [17,33,34].

When comparing the three classifiers, it is evident that our proposed HRGNN model outperforms the traditional classifiers in a variety of scenarios. This advantage arises from two key factors. First, HRGNN is a versatile graph neural network (GNN) with robust feature extraction capabilities. Second, our method effectively extracts hierarchical features from brain regions, further enhancing classification performance. The classification results presented in Table 2 clearly demonstrate the superiority of our framework.

### 5.3. Ablation Study

It is essential to highlight that the graph pooling operation in the HRGNN model includes a hyperparameter, specifically the pooling parameter *p*, which significantly impacts the performance of downstream tasks. A high *p* may retain excessive unnecessary and irrelevant information, while a low *p* could result in the loss of critical information. To identify the optimal *p* value, we conducted ablation experiments within the range of [10, 20, 30, 40, 50, 60, 70, 80].

**Figure 3:**
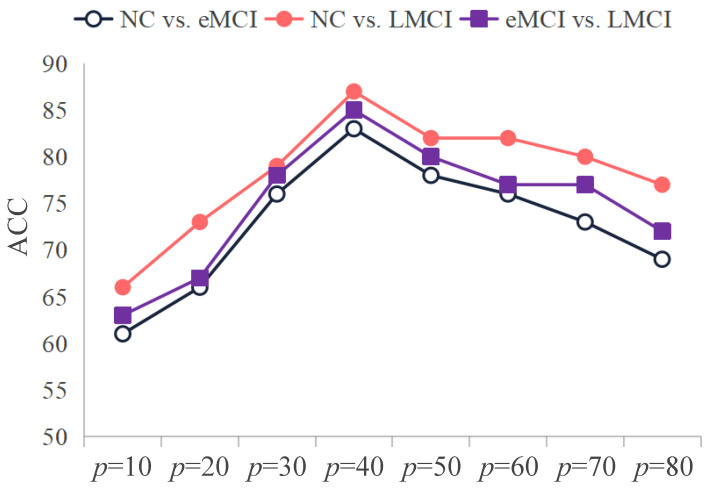
The impact of various *p* values on the classification performance of HRGNN across three classification tasks.

Figure 3 illustrates how recognition performance varies as the value of *p* is adjusted across three tasks. As depicted in Figure 3, the HRGNN model’s recognition performance exhibits a distinct pattern in these tasks. As *p* increases from 10 to 40, the model’s performance steadily improves, reaching its peak at p=40. However, as *p* continues to rise beyond this point, performance gradually declines. This indicates that p=40 represents an optimal balance. When comparing the three trends, we observe that the decline in recognition performance is relatively minor for the task that contrasts normal controls (NCs) with patients experiencing late mild cognitive impairment (LMCIs) when *p* ranges from 40 to 80. This may be attributed to the significant differences between these two groups. Although very high *p* values could introduce irrelevant information, the substantial disparities between the groups mitigate the negative impact on subsequent classification.

In summary, the ablation experiments demonstrate the significant impact of varying *p* values on the recognition performance of the HRGNN model. Specifically, setting *p* to 40 enables the HRGNN model to retain sufficient information for the subsequent task.

## 6. Discussion

### Most Discriminative Patterns

**Figure 4:**
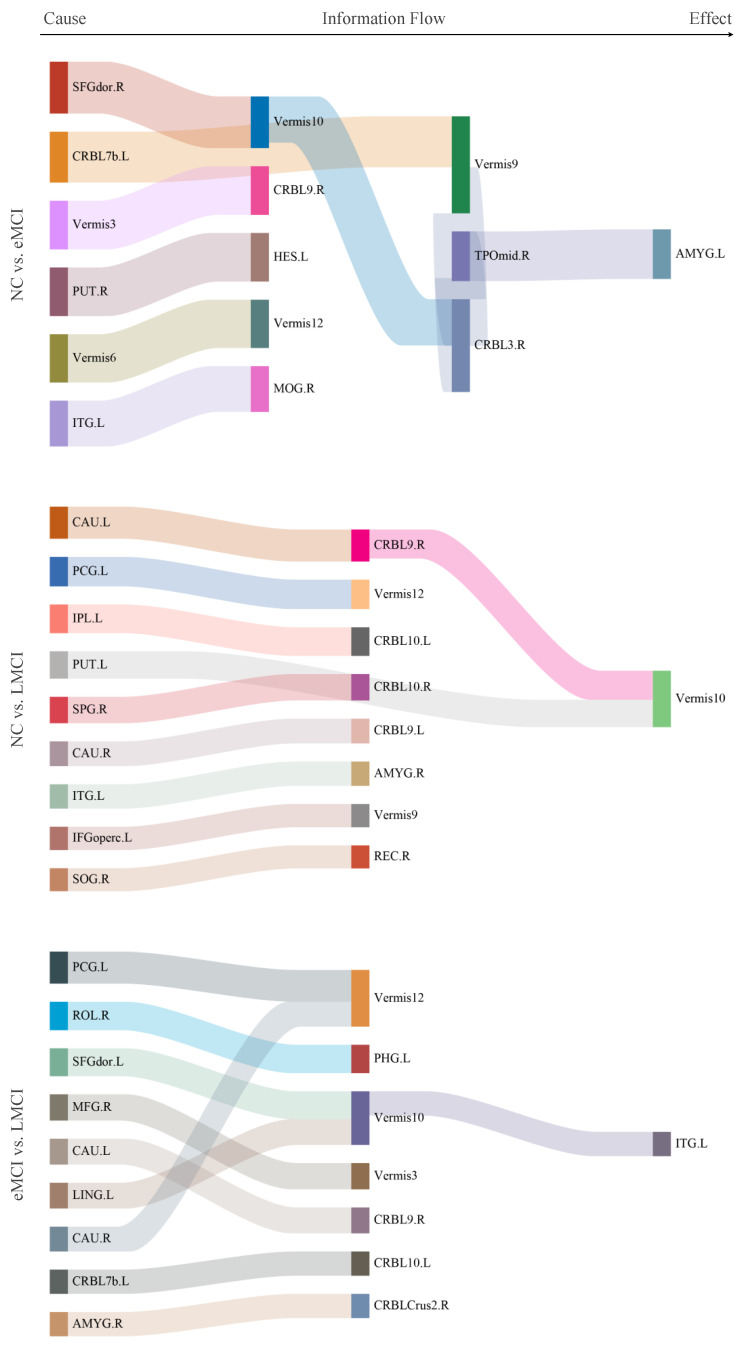
The discriminative connectivity patterns for different tasks (from left to right: NC vs. eMCI, NC vs. LMCI, and eMCI vs. LMCI).

Figure 4 illustrates the most distinctive connectivity patterns across the three tasks. The Sankey diagram displays the top 10 weighted connections identified by the SVM classifier. Here are three important aspects to consider when interpreting the circos plot. The color of each line in Figure 4 is randomly assigned only for better visualization. From Figure 4, we observe that several brain regions highlighted in the circos plot correspond with previous research findings. For instance, the inferior temporal gyrus [35,36], lenticular nucleus [35,36,37], and amygdala [38,39] have consistently been reported to have strong associations with dementia. Furthermore, our results suggest that Vermis10 may also be closely linked to the development of dementia as abnormalities in its connections were noted across all three tasks.

## 7. Limitations and Future Work

Although the BCEN model proposed in this study effectively improves diagnostic accuracy for MCI patients and may and can more accurately infer directional influences between brain regions, it still has certain limitations that present opportunities for future research. Specifically, these limitations include the following two points:

### 7.1. Interpretability of Model Results

While the proposed method, BCEN, demonstrates excellent performance in inferring directional influences between brain regions, its inherent “black-box” nature presents challenges in precisely interpreting how the model reaches specific conclusions. Although this study has provided some interpretability through weight visualization, this approach remains insufficient for fully understanding the model’s decision-making process. Future work will focus on employing more advanced interpretability techniques, such as saliency mapping, layer-wise relevance propagation (LRP), or attention mechanisms, to clearly identify which parts of the input data contribute most significantly to the prediction results. This will enhance both the credibility of the model’s findings and the depth of neuroscientific interpretation.

### 7.2. External Validation

The findings of this study are based solely on the ADNI dataset, and their generalizability remains to be validated using additional independent populations and datasets. The model’s ability to generalize is crucial for assessing its practical value. Future research will focus on externally validating the model developed in this study using other large-scale public databases (such as AIBL, OASIS, NACC, etc.) to determine whether the findings are applicable across different scanning devices, acquisition protocols, and population samples. Additionally, we will further investigate the correlations between the identified abnormal connections and specific clinical indicators (such as ADAS-Cog and CDR-SB scores), and biomarkers (such as Aβ and pTau levels). This work will facilitate the transition of the method proposed in this study from an analytical tool to one with potential clinical applications.

## 8. Conclusions

The existing algorithms employ a pairwise matching technique to estimate directional influences between brain regions. However, this approach often neglects the potential influence of other brain regions on the results. To address this limitation, we propose a novel network model that considers all the relevant brain regions when assessing directional influences. Compared to the existing methods, our approach demonstrates superior performance across various tasks with different classifiers, highlighting its effectiveness. Furthermore, this study validates commonly reported biomarkers and uncovers new ones, such as Vermis10, offering fresh perspectives and insights into related research areas. In the future, we will concentrate on optimizing the proposed network model by integrating multimodal neuroimaging data (e.g., fMRI combined with EEG or DTI) to improve the accuracy of directional influences across brain regions. Additionally, we will systematically evaluate the model’s robustness against noise and individual variability.

## Figures and Tables

**Table 1:** Demographic information about ADNI.

Group	NC	eMCI	LMCI
Male/Female	28/39	32/45	50/20
Age (mean ± STD)	74.1 ± 6.2	71.2 ± 6.9	71.2 ± 8.3

**Table 2:** Recognition performance of all methods across three different classifiers. The asterisk in the upper right corner of the value indicates a significant difference between the results of the proposed method and the suboptimal comparison method.

Classifier	Model	NC vs. eMCI				NC vs. LMCI				eMCI vs. LMCI			
		ACC	SEN	SPE	F1	ACC	SEN	SPE	F1	ACC	SEN	SPE	F1
RF	PC	69.44	67.16	71.43	67.16	76.64	71.64	81.43	75.00	74.83	76.62	72.86	76.13
	SBN	72.22	62.69	80.52	67.74	75.18	73.13	77.14	74.24	65.31	72.73	57.14	68.71
	LRBN	80.56	76.12	84.42	78.46	78.10	74.63	81.43	76.92	78.23	84.42	71.43	80.25
	SLRBN	69.44	59.70	77.92	64.52	78.10	77.61	78.57	77.61	64.63	76.62	51.43	69.41
	GC	80.56	70.15	89.61	77.05	81.75	77.61	85.71	80.62	65.31	68.83	61.43	67.52
	mGC	79.17	68.66	88.31	75.41	82.48	79.10	85.71	81.54	62.59	72.73	51.43	67.07
	PDC	75.69	77.61	74.03	74.82	78.83	73.13	84.29	77.17	69.39	71.43	67.14	70.97
	DTF	76.39	74.63	77.92	74.63	80.29	76.12	84.29	79.10	70.75	74.03	67.14	72.61
	TE	75.00	73.13	76.62	73.13	79.56	77.61	81.43	78.79	70.07	75.32	64.29	72.50
	Ours	81.25	76.12	85.71	79.07	83.21	77.61	88.57 *	81.89	76.87	85.71	67.14	79.52
SVM	PC	70.14	67.16	72.73	67.67	74.45	70.15	78.57	72.87	67.35	64.94	70.00	67.57
	SBN	75.00	70.15	79.22	72.31	77.37	74.63	80.00	76.34	70.07	81.82	57.14	74.12
	LRBN	82.64	79.10	85.71	80.92	83.94	79.10	88.57	82.81	81.63	85.71	77.14	83.02
	SLRBN	73.61	70.15	76.62	71.21	76.64	74.63	78.57	75.76	65.31	81.82	47.14	71.19
	GC	80.56	79.10	81.82	79.10	80.29	79.10	81.43	79.70	69.39	74.03	64.29	71.70
	mGC	81.94	74.63	88.31	79.37	82.48	85.07	80.00	82.61	80.95	77.92	84.29	81.08
	PDC	75.69	77.61	74.03	74.82	79.56	76.12	82.86	78.46	71.43	75.32	67.14	73.42
	DTF	77.08	76.12	77.92	75.56	81.02	77.61	84.29	80.00	72.79	75.32	70.00	74.36
	TE	77.08	77.61	76.62	75.91	80.29	77.61	82.86	79.39	73.47	74.03	72.86	74.51
	Ours	83.33	79.10	87.01	81.54	85.40 *	82.09	88.57	84.62 *	82.99 *	87.01 *	78.57	84.28
HRGNN	PC	75.69	73.13	77.92	73.68	76.64	82.09	71.43	77.46	71.43	66.23	77.14	70.83
	SBN	78.47	71.64	84.42	75.59	78.10	82.09	74.29	78.57	75.51	84.42	65.71	78.31
	LRBN	79.86	76.12	83.12	77.86	81.02	76.12	85.71	79.69	79.59	84.42	74.29	81.25
	SLRBN	73.61	71.64	75.32	71.64	74.45	68.66	80.00	72.44	71.43	72.73	70.00	72.73
	GC	82.64	80.60	84.42	81.20	81.02	76.12	85.71	79.69	77.55	80.52	74.29	78.98
	mGC	81.94	77.61	85.71	80.00	83.21	79.10	87.14	82.17	78.23	83.12	72.86	80.00
	PDC	77.78	79.10	76.62	76.81	81.02	74.63	87.14	79.37	72.79	75.32	70.00	74.36
	DTF	79.86	79.10	80.52	78.52	80.29	76.12	84.29	79.10	74.83	74.03	75.71	75.50
	TE	78.47	76.12	80.52	76.69	81.75	77.61	85.71	80.62	75.51	75.32	75.71	76.32
	Ours	84.03 *	82.09 *	85.71	82.71	84.67	82.09	87.14	83.97 *	83.67 *	85.71	81.43 *	84.62 *

## Data Availability

Data available in a publicly accessible repository.
